# Supplementary material for: A high-resolution mRNA expression time course of embryonic development in zebrafish
Source: eLife. 2017 Nov 16;6:e30860. doi: 10.7554/eLife.30860 (PMC5690287; doi:10.7554/eLife.30860)
Supplement: Supplementary file 6. [file elife-30860-supp6.zip › biolayout-clusters-files/Cluster122-genes.html]

Cluster122


# Cluster122: Genes

| Ensembl ID | Gene Name | Chr | Start | End | Biotype |
| --- | --- | --- | --- | --- | --- |
| ENSDARG00000104295 | arhgap21a | 24 | 6481872 | 6617946 | protein\_coding |
| ENSDARG00000079549 | cdc42ep1a | 12 | 19118736 | 19128980 | protein\_coding |
| ENSDARG00000101413 | dusp7 | 11 | 132684 | 148096 | protein\_coding |
| ENSDARG00000012586 | fmnl2a | 9 | 4764057 | 4890674 | protein\_coding |
| ENSDARG00000079939 | jmjd1cb | 12 | 8544032 | 8780156 | protein\_coding |
| ENSDARG00000076075 | myh7ba | 11 | 24945111 | 24976035 | protein\_coding |
| ENSDARG00000078145 | si:ch211-218g4.2 | 23 | 15290361 | 15442072 | protein\_coding |
| ENSDARG00000103181 | si:dkey-34d22.1 | 16 | 35391133 | 35424226 | protein\_coding |
| ENSDARG00000000370 | triob | 16 | 8853370 | 9108908 | protein\_coding |
| ENSDARG00000055106 | znf148 | 9 | 38814683 | 38831512 | protein\_coding |
| ENSDARG00000062231 | znf362b | 23 | 20812039 | 20837884 | protein\_coding |
